# Who Is at Risk for Stillbirth? The Discussion Continues

**DOI:** 10.1111/ppe.13179

**Published:** 2025-02-05

**Authors:** Robert W. Platt

**Affiliations:** ^1^ Department of Epidemiology Biostatistics, and Occupational Health McGill University Montreal Canada

1

The debate over denominators for gestational age‐specific analyses has challenged perinatal epidemiology for almost 40 years. Yudkin et al. [1] first proposed that stillbirths be counted as a function of foetuses in utero, a method expanded on by Joseph et al. [2] and others into what we now know as the ‘fetuses at risk’. This has led to extensive debate and discussion in the literature on choices of denominators, including several papers in this journal [3, 4, 5]. In this issue of *Paediatric and Perinatal Epidemiology*, Sexton et al. [6] study trends in gestational age‐specific stillbirth in Australia with three different denominators: all births at a specific gestational age, all foetuses at risk and continuity‐corrected foetuses at risk measure.

This ongoing debate raises several questions. The first such question is, why do we need denominators in the first place? We use denominators to estimate risks and rates (note that the stillbirth ‘rate’ is not really a rate as there is no person‐time under consideration unless we consider gestational age as a time scale [7]). The risk of stillbirth should be the ratio of the number of stillbirths among those at risk and the number at risk; therefore, the critical question in creating a gestational age‐specific risk is, who is at risk? Following Yudkin, it seems logical that foetuses are at risk for stillbirth, and the foetuses at risk denominator (or variations thereon) is the appropriate one.

Why do we do gestational age‐specific analyses for stillbirth at all? As Sexton et al. point out, we may want to know about trends in gestational age‐specific stillbirths, and we may wish to focus on certain gestational ages to assess the rate of preventable stillbirths better. Cross‐national comparisons may be helped by looking at gestational age‐specific stillbirth rates because the standardisation by gestational age allows comparisons that account for differences between populations in the gestational age distribution. These primarily descriptive analyses help understand population differences and perhaps identify critical windows for interventions.

It is worth reminding ourselves of the principal issue that was raised by Schisterman and Sjaarda [3] in a commentary in this journal. What is the question we are trying to answer by producing these rates? If, as noted above, we are simply interested in presenting the pattern of stillbirth rates over gestational age or describing population differences, the foetuses at risk measure of stillbirth is probably the most appropriate measure. As Sexton et al. show, it has appealing properties, but more importantly, as Yudkin described, it is foetuses that are actually at risk for stillbirth. Once a baby has been delivered, it is no longer at risk, so to count them as part of the denominator does not make sense. It is hard to think of a setting where the gestational age‐specific stillbirth rate, which uses total births at a given gestation, is relevant to this study.

However, once a comparative (i.e., causal) question is of interest, the choice of denominator becomes more complicated, and as others have made clear, the denominator should be driven by the question. First, in many cases, delivery is downstream from exposure (e.g., for early pregnancy exposures, the at‐risk cohort is defined by those exposed at the time they are exposed, not when they are delivered). Conditioning on gestational age (by stratifying the denominator), regardless of the rationale or choice of the denominator, is not appropriate, in particular when gestational age is affected by the exposure [8]; if researchers are interested in disaggregating total effects of exposure on stillbirth into the indirect mediated by gestational age and a direct effect, it is essential that appropriate methods [9] be used to account for the causal structure under study.

The target trial framework [10] may inform the choice of the denominator, too! Suppose one constructs a hypothetical trial of a disease comparing those with and without an exposure. In that case, it becomes evident that the appropriate denominator is chosen as a function of the exposure and the design of the hypothetical target trial, not gestational age. For early pregnancy exposure, the denominator is naturally all those ongoing pregnancies that are exposed to the treatment and control.

In conclusion, while I appreciate Sexton et al.'s effort to find the ‘best’ denominator for the analysis of stillbirth, I would like to ensure that appropriate focus is given to the research question and why we choose one denominator over another.

## Disclosure

The author has nothing to report.

## Conflicts of Interest

The author declares no conflicts of interest.

## Data Availability

The author has nothing to report.
